# A prospective follow-up study of first-episode acute transient psychotic disorder in Latvia

**DOI:** 10.1186/1744-859X-13-4

**Published:** 2014-02-06

**Authors:** Marija Rusaka, Elmārs Rancāns

**Affiliations:** 1 Riga Centre of Psychiatry and Addiction Disorders, Tvaika str. 2, Riga LV 1005, Latvia; 2 Department of Psychiatry and Addiction Disorders, Riga Stradins University, Tvaika str. 2, Riga LV 1005, Latvia

**Keywords:** Acute and transient psychotic disorder, First episode psychosis, Clinical features, Stressful life event

## Abstract

**Background:**

Acute and transient psychotic disorder (ATPD) has been described as an acute psychosis with brief onset and polymorphous symptomatology (WHO, 1993). The study of ATPD is growing increasingly relevant as scientists start an active discussion of the possibility of changing the ATPD classification in the next International Classification of Diseases (ICD-11). The aims of this study were to describe the clinical features of the index episode of ATPD in patients in Latvia, to analyse the stability and longitudinal changes of the diagnosis, to explore potential correlations between the sociodemographic and disease characteristics and to describe stressful life events before the first ATPD episode.

**Methods:**

A prospective follow-up study of all first-time admitted patients from the Riga Centre of Psychiatry and Addiction Disorders who fulfilled the ICD-10 criteria for ATPD (WHO, 1993) during the 15-month period from 9 January 2010 to 30 March 2011 and followed up until 31 October 2012. Stressful life events, demographics and clinical features during the index episode were assessed.

**Results:**

One hundred two patients were admitted with first-episode ATPD. The majority were females (60.7%). Over an average 26.5-month follow-up period, 59.8% of the patients were not readmitted. The overall stability rate of ATPD diagnosis in our sample was 67.4% (*p* = 0.0001). In the subgroup of patients who were readmitted, 70.7% had their diagnosis converted to schizophrenia in subsequent visits. Stressful life events before the first episode were found in 49.0% of first-episode ATPD patients. Thought disorder was found to be the strongest statistically significant predictor of ATPD diagnosis conversation to schizophrenia (odds ratio 4.3), with high Wald's criterion (9.435) in binary logistic regression.

**Conclusions:**

ATPD is prevalent in Latvia, with rather high overall stability rate. Combining these data from first-episode ATPD patients in Latvia with data from other countries may help predict the development of the disease and provide a basis for potential changes to ICD-11.

## Background

Acute and transient psychotic disorder (ATPD; F23) was added to the WHO International Classification of Diseases (ICD-10) as a separate syndromalogical unit in 1992 [1]. ATPD is a disorder characterised by acute onset, psychotic symptomatology and rapid resolution. There are relatively few large epidemiological studies that employ standardised assessment methods to investigate this disorder. This leaves important clinical questions unaddressed. We lack sufficient information on the long-term diagnostic stability of acute psychosis, epidemiological and clinical characteristics, the association of the disorder with stress, and on its relationship with schizophrenia [2,3].

However, the popularity of ATPD studies has increased sharply over the last few years. From 2009 through 2011, only a few articles appeared in the USA National Library of Medicine National Institutes of Health (PubMed) database [4-10]. In 2012, many new studies on the topic were published [11-18]. This increased research attention may be due to the active debate over ATPD's place in the next revision of the International Classification of Diseases, ICD-11.

International study results show that the prevalence of the disorder is very variable [2]. In the UK, prevalence was reported at 3.9 cases per 100,000 of the population, while in Denmark, prevalence was reported to be 9.6 [3,19]. In 2009, the prevalence of ATPD in Latvia was reported as high as 10 per 100,000 populations via official statistics [20]. The overall stability rates of ATPD diagnosis in follow-up studies ranged from 34.0% to 73.0% (follow-up period between 1 and 5 years) and converted mainly to either F2 schizophrenia and related disorders or to F3 affective disorders [2,4,14,21].

Stressful life events have been associated with an increased risk of mental disorders. There have been few studies examining the specific connection between stressful life events and ATPD [2]. Raune et al. concluded that stressful events, especially in the preceding 3 months, may trigger the first episode of ATPD psychosis [22]. Chakraborty et al. demonstrated that stressful life events occur more frequently in the 6 months prior to the onset of an ATPD episode, as compared to the 6 months before to the onset of a manic episode [23]. Thus, there is no sufficient information available regarding the epidemiology, clinical characteristics and prognosis of ATPD.

### The aims

The aims of this study were to describe the clinical features of the index episode of ATPD in patients in Latvia, to analyse the overall stability and longitudinal changes of the diagnosis and to explore potential correlations between the sociodemographic characteristics and disease characteristics. In addition, we describe stressful life events before the first ATPD episode.

## Methods

In this prospective follow-up study, we identified all patients who were admitted for the first time with a diagnosis of ATPD according to ICD-10 [1] at the Riga Centre of Psychiatry and Addiction Disorders (RCPAD), Latvia, during a 15-month period (from 9 January 2010 to 30 March 2011). The clinical diagnosis was re-evaluated independently by the authors using the ICD-10 Diagnostic Criteria for Research for ATPD [1].

Demographics and clinical features during the index episode were assessed as in Marneros and Pillmann [2]. To analyse the longitudinal changes in the ATPD diagnosis group, the patients were followed up until 31 October 2012. Stressful life events (SLE) were assessed using methods in the HASAP study, which is in accordance with the criteria generally used in life event research. Events occurring during the 6 months prior to the index episode were considered [2]. Eight major negative stressful life events: death of significant other, separation/divorce, serious illness/operation, serious problems at work, change of job/school, major journey, relocation of residence and serious problems at family were assessed as described in the Holmes and Rahe study [24]. To determine the personality profile for each patient, the Mini-Mult scale by Kincannon, the short version of The Minnesota Multiphasic Personality Inventory (MMPI) scale, was used 1–3 days before discharge from initial admission [25-27].

The Mini-Mult scale consists of 71 items from 11 of the 13 standard MMPI scale [25]. The Mini-Mult contains three rating scales (a Scale of Lie (L), a Scale of Integrity (F), and a Scale of Correction (K)) and eight basic scales (Hypochondry (Hs), Depression (D), Hysteria (Hy), Psychopathy (Pd), Paranoid (Pa), Psychasthenia (Pt), Schizoid (Se), and Hypomania scales (Ma) [25].

For the purpose of comparison, the patients were divided into two groups. The first group, designated the ‘pure’ ATPD patient group, included all patients who were not readmitted and any patient who was later readmitted with a diagnosis of ATPD. The second group consisted of patients who were readmitted with a diagnosis of schizophrenia (F20, ICD-10) [1].

Statistical analyses were performed using the Statistical Package for Social Sciences (SPSS), version 19.0. To assess the difference between the two groups, for parametric data, Mann–Whitney *U* test was used for two-sample comparison. Proportional to the normal distribution of data consistency was determined using the Kolmogorov-Smirnov test. Qualitative differences in the patient population were evaluated using the Pearson's chi-square (*χ*^2^) or Fisher's exact test.

An association between the variables was used for nonparametric Spearman rank correlation test. In order to determine the potential impact of external factors on the subsequent development of the patients' diagnoses, all demographic variables, clinical features, SLE and personality profile data was included in the logistic regression analysis. For selected data with statistically significant *p* value (less than or equal to 0.05.), binary logistic regression (Wald test) was used. The study protocol was approved by the Riga Stradins University Ethics Committee.

## Results

**Figure 1:**
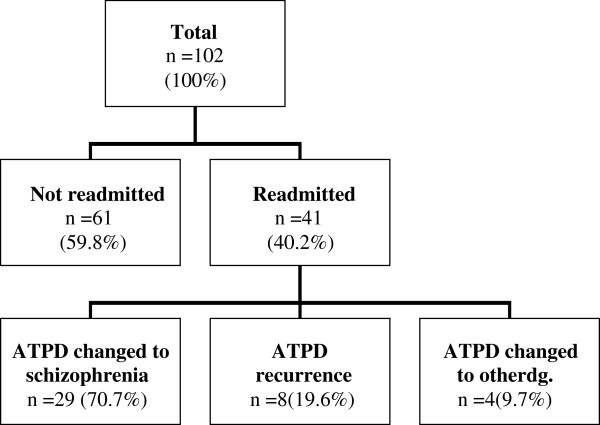
Longitudinal changes of diagnosis of the study group.

During a 15-month period, 102 patients were admitted with a first-time diagnosis of ATPD. The majority were females (60.7%, *n* = 62). The average age at diagnosis of the first psychotic episode for females was 40.2 (SD = 13.4; 95% Cl ± 6.9), and for males was 29.0 (SD = 10.2; 95% Cl ± 6.5) (*p* < 0.0001). Only 15.7% (*n* = 16) of the patients had a family history of psychiatric disorders. A quarter (*n* = 25) of patients had a general secondary education, 34.3% (*n* = 28) had a vocational secondary education and 25.4% (*n* = 26) had completed a higher education. Approximately one third (30.3%, *n* = 31) of the patients were married. Patients with a ‘pure’ ATPD diagnosis were significantly more likely to be married than the other patients and comprised 80% (*n* = 25) of the married patient group (*p* = 0.0001). During their first psychotic episode, the patients were treated in the hospital an average of 21.6 hospital bed days (SD = 9.9, 95% CI ± 3.9). The group of patients whose ATPD diagnosis was later changed to schizophrenia were hospitalised longer during their first psychotic episode than the patients from the ‘pure’ ATPD group (20.0 vs. 26.4 bed days, *p* = 0.004). In the follow-up period, averaging 26.5 months, 59.8% (*n* = 61) of the patients were not readmitted. The longitudinal changes of diagnosis are presented in Figure 1. The overall stability rate of ATPD diagnosis in our sample reached 67.4% (*p* = 0.0001).

### Clinical features and initial diagnosis

**Figure 2:**
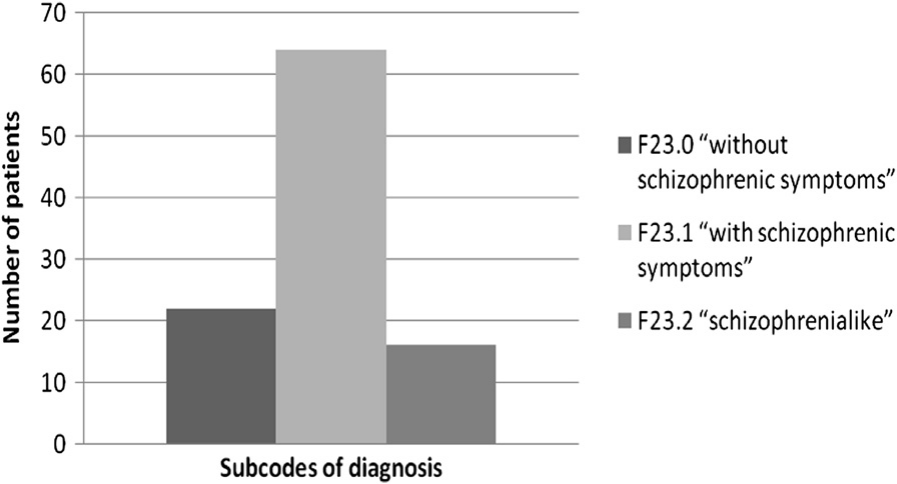
The distribution of initial diagnosis.

**Figure 3:**
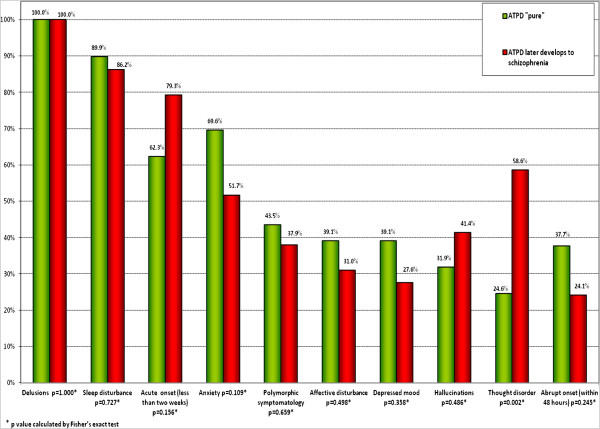
Symptomatology of the first ATPD episode.

The distribution of the initial diagnosis is described in Figure 2. A diagnosis of ATPD ‘without schizophrenic symptoms’ (F23.0) was significantly more common amongst ‘pure’ ATPD patients (63.7%; *p* = 0.01). The clinical features of index ATPD episode are presented in Figure 3. The frequency of thought disorder was statistically significant in 24.6% of the ‘pure’ ATPD patients and in 58.6% of patients with ATPD that later developed schizophrenia (*p* = 0.002).

### Life events prior to diagnosis

**Figure 4:**
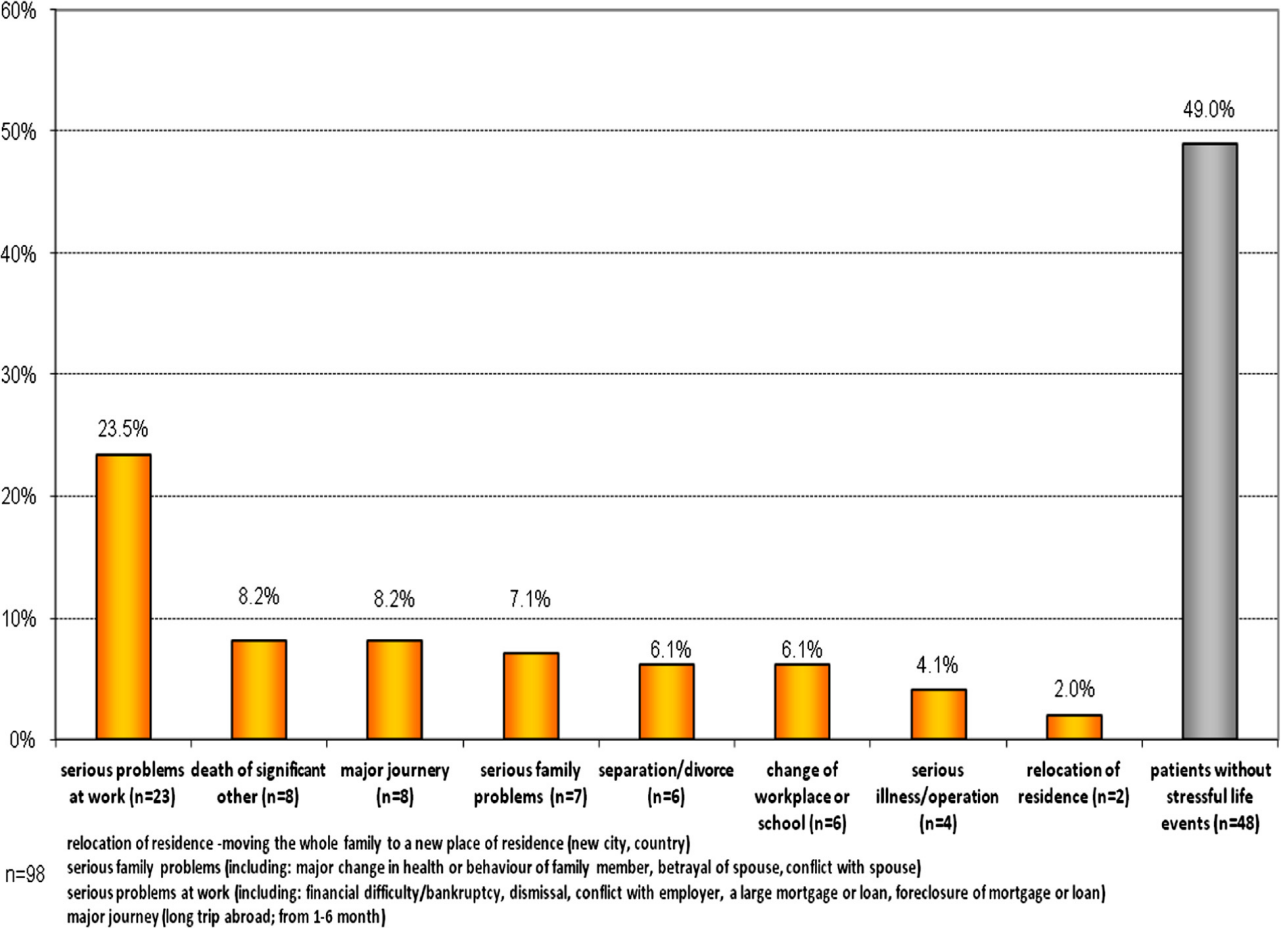
Stressful life events within 6 months before the first episode.

Associated acute stress as defined by ICD-10 was seen in only 1.0% (*n* = 1) of patients. Stressful life events that occurred in the 6 months prior to the index episode were found for 51.0% (*n* = 50) of the patients. The most common negative life events are reported in Figure 4. There were no statistically significant differences between the groups (‘pure’ ATPD and ATPD that later developed schizophrenia) for any of the stressful life events.

### Personality profiles

**Figure 5:**
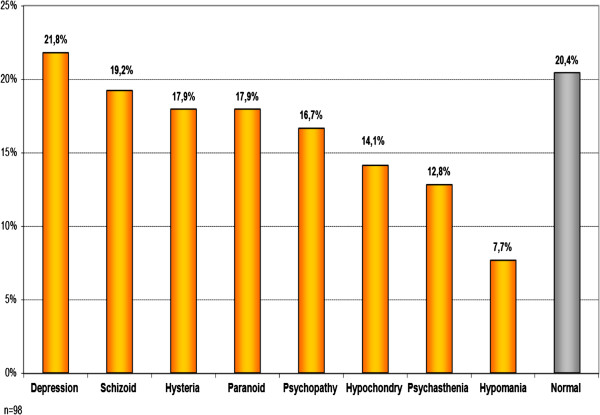
Personality profiles.

Almost a fifth of the patients, (*n* = 18, 17.6%) had a personality profile within the norm, and among these patients, those with a ‘pure’ ATPD diagnosis (*n* = 14, 77.8%) were more likely (*p* = 0.0006) to have a personality profile within the norm. The results are presented in Figure 5.

### Regression analysis

In the binary logistic regression (Wald test), thought disorder was found in 24.6% of the ‘pure’ ATPD patients and in 58.6% of patients with ATPD that later developed schizophrenia. The difference was statistically significant by both Pearson chi-square and Fisher's exact test (*p* = 0.002). There was a statistically weak correlation between the two variables (Spearman correlation value 0.326), but thought disorder was a statistically significant ‘predictor’ of ATPD diagnosis conversation to schizophrenia (in binary logistic regression, Wald's criterion was high 9.435).

## Discussion

In recent years, ATPDs have received increased attention in the psychiatric research setting. This attention may be prompted by an opportunity to improve classification during the revision of the Eleventh International Classification of Disease (ICD-11). To contribute to this effort, the aims of our study were to describe the clinical features of the index episode of ATPD in patients in Latvia, to analyse changes in the diagnosis longitudinally and to explore potential correlations between the sociodemographic characteristics of patients and their disease characteristics. We also tried to identify the longitudinal diagnostic stability of ATPD diagnosis in Latvia and correlate stressful life events before the first episode to the occurrence of the characteristics of the progression of the disease. We hope that our study results can help predict the development of disease and inform potential changes to ICD-11.

The sample at RCPAD can be regarded as a representative sample of the clinical inpatient population with ATPD in Latvia, as the hospital is the largest hospital in the country (with approximately 440 beds) and provides care for approximately 40% of the Latvian population. In a study carried out by this team in 2010, an average of 73 new ATPD cases a year were found, giving an incidence of 8.1 cases per 100,000 inhabitants [28]. This incidence is high in comparison to the ‘Nottingham’ study, which had only 3.9 cases per 100,000 population [19], but closer to a Danish cohort study which identified an incidence of 9.6 per 100,000 population [3].

In agreement with previous studies, we found a higher prevalence of ATPD in females [2,3,21,29,30]. Women composed 60.7% of the population diagnosed with ATPD in this study. This is similar to the proportion observed by Aadamsoo et al. in Estonia (60.0%) [4]. The average age at first psychotic episode in our study was higher in women than in men; this is also in agreement with previous studies, but this can be due to ‘gender X age’ effect [2].

There are only a few studies investigating the familial psychiatric morbidity of individuals with ATPD. Marneros and Pillmann reported a higher rate of mental disorders among family members of patients with ATPD than among the relatives of healthy controls, but no significantly increased frequency of psychotic disorders among these family members was found [2]. In our study, only 15.6% (*n* = 16) the patients were found to have a family history of psychiatric disorder.

We also surveyed the level of formal education among this patient cohort. Similar to results reported by Marneros and Pillman, about one quarter of the patients in this study had completed higher education [2].

Almost a third of the patients were married. Interestingly, patients with a ‘pure’ ATPD diagnosis were significantly (*p* = 0.0001) more likely to be married than the other patients and composed 80% of the married patient group. Aadamsoo et al. found that 42.3% of the ATPD patients were married compared to only 11.4% of patients with a schizophrenia diagnosis [4]. This may indicate that before the first episode of psychosis, the level of social functioning is higher among the group of patients with a ‘pure’ ATPD diagnosis than among those with a diagnosis which changes to schizophrenia.

During the first psychotic episode, the patients were treated in the hospital an average of 21.6 hospital bed days. This is similar to other ATPD studies. Interestingly, the patients whose ATPD diagnosis was later changed to schizophrenia were treated longer upon their first psychotic episode than patients from the ‘pure’ ATPD group (*p* = 0.004).

Just over 40% of patients were readmitted during the follow-up period. This could indicate a better prognosis for ATPD than for schizophrenia. If the ATPD diagnosis was changed, in 70.7% of cases, it was changed to a diagnosis of schizophrenia. This is similar to the data of Aadamsoo et al., in which 64.0% of changed ATPD diagnoses were changed to schizophrenia [4].

Although the follow-up period here averaged only 26.5 months, our previous retrospective study with a follow-up period of 6 years showed that diagnosis conversion took place within the first 2 years after initial hospitalisation for 81.0% of patients [28]. Thus, we believe that our current study data captures a significant portion of the trends in diagnosis conversion for ATPD patients.

ATPD has lower rates of relapse in developing countries as compared to industrialised countries and has a relatively high diagnostic stability in Europe [21]. The overall stability rate in our study was high (67.4%; 2.2-year follow-up period), and this is similar to those reported by Marneros and Pillman (54.0%; 4.7-year follow-up period), Jørgensen et al. (52.0%; 3-year follow-up period) and Castagnini et al. (48.4%; 5-year follow-up period) [2,14,29].

Aadamsoo et al. reported a lower stability rate at 34.0% (2-year follow-up period) [4]. This may be due to the methodological differences between our studies. We used the methods similar to those in Castagnini and Berrios, which include patients who were not readmitted after the first episode, but Aadamsoo et al. did not include those patients in their calculations [4,9].

The percentage of F23.0 patients in our study population (21.5%) was smaller than that described by Aadamsoo et al. (25.0%) in Estonia. However, the F23.1 diagnosis was much more frequent at 62.7% in the Latvian study compared to 29.0% in Estonia [4]. This finding may demonstrate differences in diagnostic interpretations by the psychiatrists in the two neighbouring countries.

Studying the changes in diagnosis longitudinally for patients with F23.0 diagnoses vs. those with F23.1 diagnoses, we determined that patients with an F23.0 diagnosis were significantly more likely to have their diagnoses remain ‘pure’ ATPD. Similar results were found in both Japan and Estonia [4,30]. This could play a significant role in the development of amendments to the new classification (ICD-11).

Some differences between the clinical features of the first episode of psychosis associated with a ‘pure’ ATPD diagnosis and those associated with ATPD which was later converted to schizophrenia were observed. The Latvian study shows that thought disorder was found in 58.6% of patients with ATPD that later developed schizophrenia (*p* = 0.002) with a statistically significant relevance (Spearman correlation value 0.326). This could be a strong statistically significant ‘predictor’ of ATPD diagnosis conversation to schizophrenia (in binary logistic regression, Wald's criterion was high 9.435). Thus, in the first-episode ATPD patients with thought disorder, the odds ratio of getting a diagnosis change to schizophrenia is 4.3 times higher than those in patients without thought disorder.

Studies of reactive psychosis (which is included in ICD-10 under ATPD diagnosis and DSM IV under brief psychotic disorder) were very popular in Scandinavia during the 1970s and 1980s [1,31-36]. All of these studies identified a correlation between the onset of ATPD and stressful life events preceding the first episode of psychosis [2,18,21,23]. We also found that a large proportion of patients in our study had experienced stressful life events during the 6 months prior to their first psychotic episode. Thus, our research supports the argument that stressful life events can be an important factor facilitating the development of this disease.

In SRRS, each one of the stressful life events is awarded a life change unit (LCU), depending on how traumatic it was felt to be by a large sample of participants. In this study, to compare SLE as a risk factor, we divided the SLE into two groups: (1) high (100–45) LCU level: death of significant other, separation/divorce, serious illness/operation and serious problems at work and (2) low (45–11) LCU level: change of job/school, major journey, relocation of residence and serious problems in family. We did not find any statistically significant difference between the groups [24]. But, perhaps, the relatively small sample size precluded finding statistically significant differences between the groups of patients.

In the literature, there is a considerable number of reports on the correlation of premorbid personality with schizophrenia; however, there is a lack of research about correlation of ATPD with premorbid personality profile [37]. Empirical data presented by Jørgensen et al. who, in a sample of 51 ATPD patients assessed with the International Personality Disorder Examination (IPDE) and found relatively high prevalence of personality disorders and ATPD [38].

Pillmann et al. in their study used the Neuroticism-Extroversion-Openness Five Factor Inventory (NEO-FFI) self-rating scale. They found no differences between healthy controls and ATPD patients (*n* = 42) [39]. We used the ‘Mini-Mult’ scale by Kincannon, which assesses the personality profile more objectively than the NEO-FFI used in other studies [25]. In our study, 17.6% had a personality profile within the norm and were from the ‘pure’ ATPD diagnosis group. This may indicate that before the first episode of psychosis, the level of social functioning is higher among the group of patients with a ‘pure’ ATPD diagnosis than among those with a diagnosis which changes to schizophrenia.

A large portion of ATPD patients showed deviations from the norm in personality profiles, similar to that found by Jørgensen et al., where 63% of ATPD patients also had a prevalence of personality disorders [38]. The Latvian results are similar those of other studies, but when we tried to compare ‘Mini-Mult’ scores of the ‘pure’ ATPD group and the group whose diagnosis later converted to schizophrenia, we could not find any statistically significant difference [38].

### Limitations

The limitations of this study are similar to those of other studies assessing. No structured life event scale was used, and life event data may have been influenced by recall bias, as was demonstrated in the ‘Nottingham’ study [19]. One of the most important limitation is possible bias due to differences in interview methods, sampling or clinical assessment which will affect diagnostic stability and outcomes for ATPD subtype results [4]. The limitations of the personal profile results are intrinsic to the ‘Mini-Mult’ method. Some authors argue that the ‘Mini-Mult’ shows success in screening, but the full MMPI is preferable for screening descriptive features, and personality assessment of patients occurs after the first psychotic episode [40]. In the Latvian study, the sample size was rather small, and the follow-up period was shorter than those in other studies. Due to this, the study may be underpowered for finding statistically significant differences between the groups of patients.

## Conclusions

More than half of the total patient population was admitted only once during the study period. In the subgroup of readmitted patients, the most common diagnostic change was conversion to schizophrenia. The clinical features at the first episode of the disease, such as thought disorder, could help predict further development of the disease. Stressful life events before the first episode occurred in a large proportion of ATPD patients. In the future, it is necessary to analyse a larger group of patients to perform a more sophisticated analysis of predictive clinical data.

## Competing interests

The authors declare that they have no competing interests.

## Authors’ contributions

MR was the co-designer of the study, collected the data, performed statistical analysis, interpreted the data, and wrote the manuscript. ER carried out the design of the study, participated in the statistical analysis, and has contributed substantially to the interpretation of the data and critical revision of the manuscript. All authors read and approved the final manuscript.
